# A 53‐year‐old woman with a rapidly progressive, non‐enhancing left frontotemporal lesion

**DOI:** 10.1111/bpa.13125

**Published:** 2022-11-08

**Authors:** Michael L. Miller, Daniel W. Griepp, Yu Sun, Sean Tamir, Rebecca Straus Farber, Marc L. Otten, Osama Al‐Dalahmah

**Affiliations:** ^1^ Department of Pathology and Cell Biology Columbia University Irving Medical Center New York New York USA; ^2^ Department of Medical Education New York Institute of Technology Old Westbury New York USA; ^3^ Department of Radiology Columbia University Irving Medical Center New York New York USA; ^4^ Department of Neurology Columbia University Irving Medical Center New York New York USA; ^5^ Department of Neurological Surgery Columbia University Irving Medical Center New York New York USA; ^6^ Present address: Division of Neurosurgery, Ascension Providence Hospital College of Human Medicine, Michigan State University Southfield MI 48075 USA

**Keywords:** ABRA, amyloid beta‐related angiitis, amyloid deposition, cerebral amyloid angiopathy, cerebral vasculitis

## Abstract

Fifty‐three‐year‐old woman presented with chronic, episodic headache.

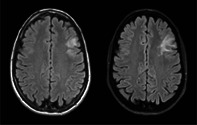

## 1 CLINICAL HISTORY AND IMAGING

**FIGURE 1:**
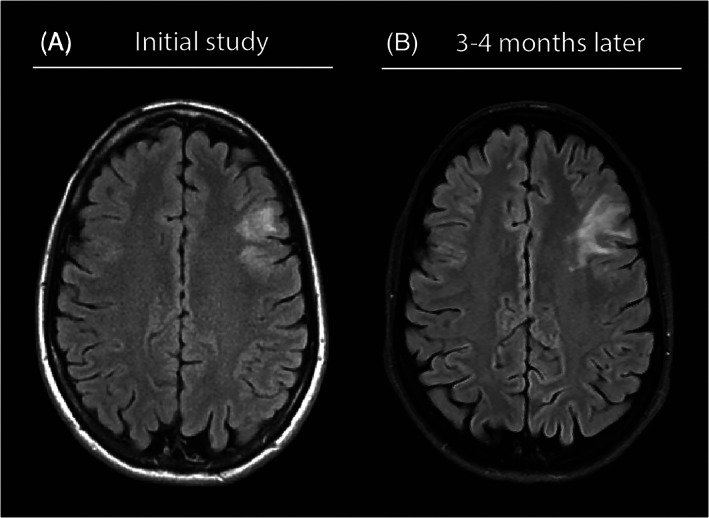
Brain magnetic resonance imaging. Axial plane FLAIR sequences at initial presentation (A) and at follow up 3–4 months later (B) revealed relatively rapid progression of the left frontal lobe.

A 53‐year‐old woman presented with chronic, episodic headache. The patient's headache was first noted several years prior to presentation and would occur for weeks to months, then remit for several weeks to months. More recently, the severity of the headache worsened which prompted a referral to neurology. The patient's past medical history included bilateral knee arthralgia and swelling about 6 years prior to presentation, which since resolved. While the symptoms raised the possibility of Lyme disease or rheumatoid arthritis (RA), neither diagnosis was confirmed. Given the patient's intractable headache, magnetic resonance imaging (MRI) was performed which revealed a non‐enhancing left frontal white matter lesion (Figure 1A). On evaluation by neurosurgery, observation was initially recommended with the possibility of open biopsy. At follow‐up, despite resolution of the patient's presenting symptom of headache, the patient began to show signs of subjective neurocognitive impairments, including word‐finding difficulty, poor performance playing chess, and fear of driving. Repeat imaging 3 months since presentation revealed progression of the lesion (Figure 1B) with expansion into the temporal lobe (not shown). Given the relatively rapid radiographic progression, the lesion was biopsied with concern for a neoplastic process (Box 1).

BOX 1 **Slide scan**

Access the whole slide scan at https://isn‐slidearchive.org/?col=ISN&fol=Archive&file=BPA‐21‐12‐302.svs

## 2 FINDINGS

**FIGURE 2:**
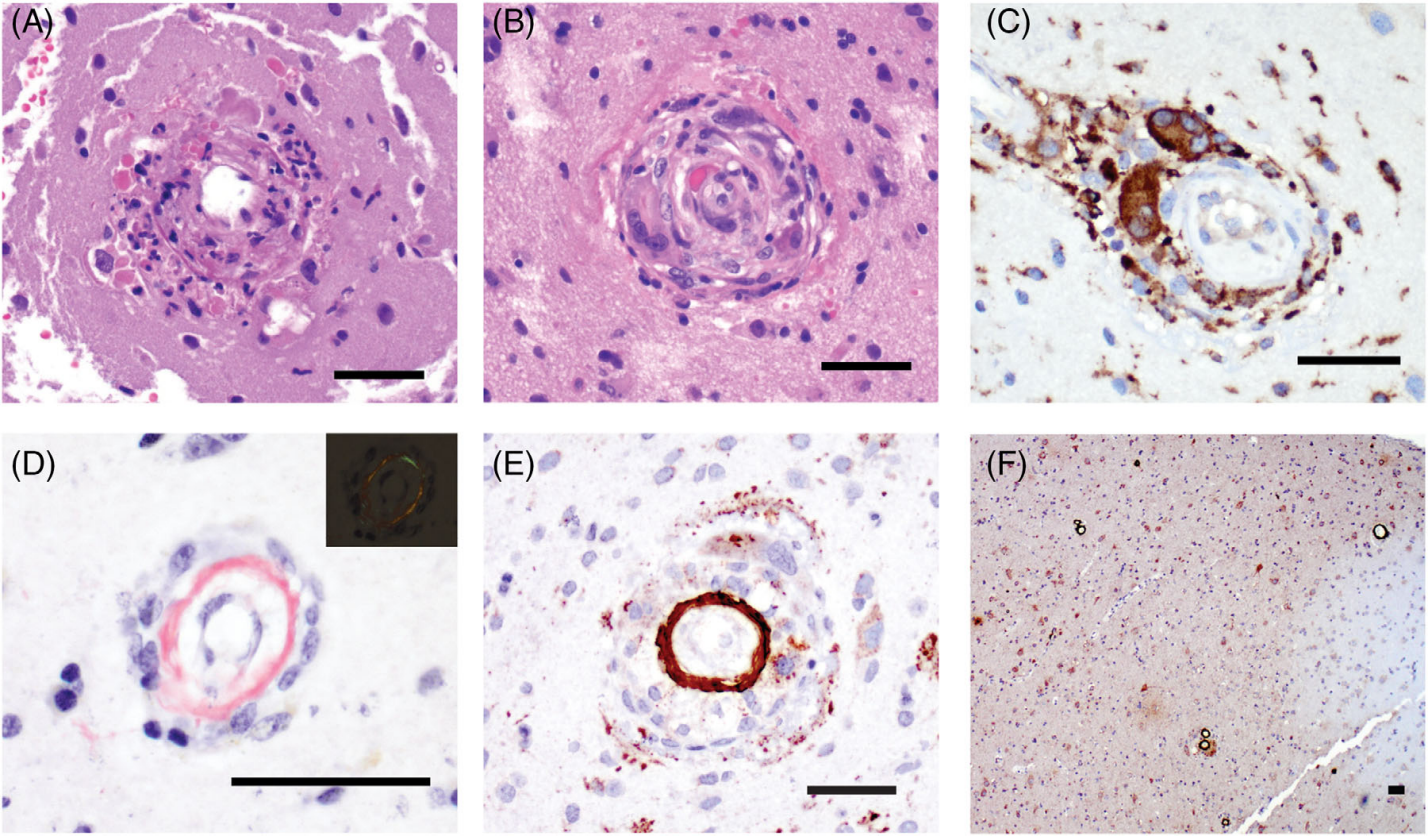
Histology of brain biopsy. Gliotic brain parenchyma with focal fibrinous exudate of vessels (A) and angiocentric inflammation composed of monocytes, lymphocytes, and prominent giant cells (B). (C) Giant cells were highlighted with CD68 immunostain. (D) Congophilic material within vascular walls, which appeared apple‐green when viewed under polarized light (inset). (E and F) Intense, circumferential accumulation of beta‐amyloid around intraparenchymal vessel, including vessels with prominent transmural inflammation (for all, scale bar = 50 μm)

Hematoxylin and eosin (H&E)‐stained sections revealed gliotic brain. Mixed chronic‐ and focally acute‐appearing inflammatory infiltrates composed primarily of histiocytes and multinucleated giant cells, with scant lymphocytes and occasional eosinophils, involved most cortical vessels (Figure 2A–C). Transmural disruption and focal necrosis were also identified **(**Figure 2A **)**, as were scattered well‐formed granulomas. Cortical and leptomeningeal vessels appeared thickened and occasionally produced a double‐barreled appearance. Focally exuberant perivascular hemosiderin deposits were identified. Within the vessel walls, deposition of amorphous, congophilic material was identified that appeared green‐red birefringent under polarized light **(**Figure 2D,E **)**. Immunohistochemistry with beta‐amyloid revealed intense circumferential staining in the leptomeningeal and cortical blood vessels **(**Figure 2F **)**.

## 3 FINAL DIAGNOSIS

Amyloid beta‐related angiitis (ABRA).

## 4 DISCUSSION

A defining feature of ABRAis the presence of cerebral amyloid angiopathy (CAA) in association with vasculitis [1]. Given prognostic and predictive differences of ABRA when compared to CNS vasculitis in the absence of CAA and CAA in the absence of vasculitis, accurate distinction is clinically meaningful [2]. For instance, in contrast to ABRA, primary CNS vasculitis without CAA generally results in a less favorable outcome [2]. While CAA is primarily the result of imbalanced amyloid production and clearance, ABRA is thought to occur when beta‐amyloid vascular deposits are recognized as foreign antigens [1]. Although ABRA generally occurs earlier in life compared to CAA, most studies describe presentation of ABRA in the sixth or seventh decades of life [1, 3]. Predisposing factors to the development of ABRA are not clearly defined, although it is associated with preexisting autoimmune processes, prior radiation treatment, and ApoE4 genotype. It is notable that the present patient was relatively young and had a possible history of rheumatoid arthritis.

The inflammatory process of ABRA results in the destruction of vessels. Previously reported cases of ABRA were typically characterized by severe leptomeningeal and parenchymal amyloid angiopathy, along with chronic inflammation within the leptomeninges and in and around vessel walls [3]. Granulomatous inflammatory infiltrates and large multinucleated macrophages are also characteristic findings [1, 3]. Extensive fibrinoid necrosis of vessels may be identified [3]. Features characteristic of CAA may also be observed, such as hemosiderin‐laden macrophages suggestive of prior hemorrhage [3]. Many of these histopathological features were appreciated in the present case.

Diagnosis of ABRA is difficult given the generally non‐specific clinical presentation and broad radiographic diagnosis. While the signs of CAA are more likely to manifest as spontaneous lobar intracerebral hematoma or progressive dementia, the presenting signs of ABRA may include seizure, mental status change, headache, or newly recognized focal neurological deficits. Radiographic changes are also nonspecific, but typically include asymmetric subcortical white matter lesions, evidence of microhemorrhages, and leptomeningeal enhancement. The present patient's clinical presentation was non‐specific and consisted of headache that progressed to word‐finding difficulty. The radiographic presentation showed a non‐enhancing white matter lesion as well as several punctate areas of susceptibility outside of the lesion. Thus overall, prior to biopsy, there was a concern for malignancy although a diverse differential including inflammatory processes was considered.

Definitive diagnosis of ABRA is rendered through microscopic examination of a targeted brain biopsy. The most successful treatments reported include immunosuppression with prednisone and/or cyclophosphamide, although other immunosuppressive agents have been successful [1, 3]. With treatment, approximately three quarters of patients recover to some degree while approximately one quarter develops relapses [1]. While in our case, the patient's young age and the rapid radiologic progression of the lesion prompted a biopsy—which was diagnostic—it is important to remember that when ABRA/CAA is clinically suspected, the diagnosis can be made without surgery when imaging is supportive.

## AUTHOR CONTRIBUTIONS

Michael L. Miller, Daniel W. Griepp and Osama Al‐Dalahmah wrote the original draft. Yu Sun, Sean Tamir, Rebecca Straus Farber and Marc L. Otten reviewed and edited the draft.

## CONFLICT OF INTEREST

The authors declare no conflict of interest.

## ETHICS STATEMENT

All data related to this case are deidentified.

## Data Availability

Data sharing is not applicable to this article as no new data were created or analyzed in this study. The whole slide scan can be accessed at https://isn‐slidearchive.org/?col=ISN&fol=Archive&file=BPA‐21‐12‐302.svs.
